# Correction: A thermal-induced electric current from a gold electrode/porous silicon device

**DOI:** 10.1039/c8ra90086h

**Published:** 2018-12-05

**Authors:** Cheng-Yin Huang, Chin-Kai Chang, Kai-Hsin Wen, Vincent K. S. Hsiao

**Affiliations:** Department of Applied Materials and Optoelectronic Engineering, National Chi Nan University Nantou Taiwan 54561 kshsiao@ncnu.edu.tw

## Abstract

Correction for ‘A thermal-induced electric current from a gold electrode/porous silicon device’ by Cheng-Yin Huang *et al.*, *RSC Adv.*, 2017, **7**, 38677–38681.

The authors regret that the name of one of the authors (Kai-Hsin Wen) was shown incorrectly in the original article. The corrected author list is as shown above.

The Royal Society of Chemistry apologises for these errors and any consequent inconvenience to authors and readers.

## Supplementary Material

